# A passive Stokes flow rectifier for Newtonian fluids

**DOI:** 10.1038/s41598-021-89699-y

**Published:** 2021-05-13

**Authors:** Aryan Mehboudi, Junghoon Yeom

**Affiliations:** 1 Department of Mechanical Engineering, The University of Texas, Austin, TX 78758 USA; 2 Code 6354, Multifunctional Materials Branch, Materials Science and Technology Division, Naval Research Laboratory, Washington, DC, 20375 USA

**Keywords:** Mechanical engineering, Fluid dynamics

## Abstract

Non-linear effects of the Navier–Stokes equations disappear under the Stokes regime of Newtonian fluid flows disallowing a flow rectification behavior. Here we show that passive flow rectification of Newtonian fluids is obtainable under the Stokes regime of both compressible and incompressible flows by introducing nonlinearity into the otherwise linear Stokes equations. Asymmetric flow resistances arise in shallow nozzle/diffuser microchannels with deformable ceiling, in which the fluid flow is governed by a non-linear coupled fluid–solid mechanics equation. The proposed model captures the unequal deflection profile of the deformable ceiling depending on the flow direction under the identical applied pressure, permitting a larger flow rate in the nozzle configuration. Ultra-low aspect ratio microchannels sealed by a flexible membrane have been fabricated to demonstrate passive flow rectification for low-Reynolds-number flows (0.001 < Re < 10) of common Newtonian fluids such as water, methanol, and isopropyl alcohol. The proposed rectification mechanism is also extended to compressible flows, leading to the first demonstration of rectifying equilibrium gas flows under the Stokes flow regime. While the maximum rectification ratio experimentally obtained in this work is limited to 1.41, a higher value up to 1.76 can be achieved by optimizing the width profile of the asymmetric microchannels.

## Introduction

Manipulating fluid flows of minute quantities is important to the field of microfluidics and lab-on-a-chip with applications spanning from microfluidic circuits^1–10^ to micropumps^11–14^ to drug delivery devices^15–18^. Among many types of devices capable of fluid flow control, fluidic diodes or rectifiers render the fluid to flow primarily in one direction analogous to the electronic counterparts^19^. They have received continuous interests thanks to their design and operational simplicity, which are mainly attributed to the *passive* nature in device operation since additional power is not needed for flow control other than the pumping power used to drive the flow. Microfluidic diodes generally focus on blocking a fluid flow in one direction^20^ and rely on moving parts such as cantilever-like flaps^21,22^, membranes^23–25^, lock-and-key microstructures^26^, and balls^27,28^ with the operating principles resembling large-scale check or one-way valves. However, these moving parts may cause reliability issues like clogging due to unavoidable adhesion to the stationary parts^29^ and lead to a potential damage of biospecies from undesirable interactions with these structures.

Microfluidic rectifiers are, on the other hand, referred to devices delivering fluid flows in both forward and backward directions but with different flow rates^20^. The simplest form of such rectifiers utilizes asymmetrical structures such as nozzle/diffuser microchannels^30–32^ and Tesla valves^33,34^, in which the absence of the moving parts simplifies device fabrication and promotes robust operation. To exhibit direction-dependent hydrodynamic resistance in the asymmetrical structures, a fluid flow requires a source of nonlinearity like the inertia terms in the Navier–Stokes equations, limiting flow rectification to fluid flows with sufficiently high Reynolds numbers (*Re* ≫ 1)^35^. The recent paper by Tao et al*.*^36^ suggests that the flow rectification or diodicity starts to diminish in asymmetric converging–diverging microchannels for the flow with the geometrically-adjusted Reynolds number less than 40. Therefore, a fluidic rectifier operating under the Stokes flow regime (*Re* < 1) entails another source of nonlinearity, most notably a use of non-Newtonian working fluids with nonlinear rheological characteristics^32,37–39^.

The recent efforts for the Stokes flow rectification of Newtonian fluids have been focused on introducing nonlinearity into the boundary conditions. For example, the surface energies or activation pressures of the channel inlets and outlets were modulated to generate asymmetrical flow resistance by means of the surface-functionalized^40^, nested^41^, or non-uniform^20,42^ nanochannels. Alvarado et al. harnessed viscoelastic pillar arrays anchored to the channel surface with tilt angles to produce nonlinear, direction-dependent drag behaviors and in turn uneven flow impedance for low *Re* flows^43^. These nonlinearities in the boundary conditions would rectify the flow even in the straight channels, i.e., asymmetrically-shaped channels not required, but the fabrication processes to induce such effects are rather involved and may not be scalable. More importantly, to our knowledge, rectification of gaseous flows with equilibrium processes (Knudsen number smaller than 0.001) has not been demonstrated in the Stokes flow regime mainly because the existing approaches are not effective for the gaseous flows.

In this paper, we present a more straightforward and universal approach to introducing nonlinear effects to the equations of motion for the Stokes flow of Newtonian fluids by leveraging the framework of shallow deformable microfluidics^44,45^. A shallow nozzle/diffuser microchannel with deformable ceiling provides a nonlinear, direction-dependent coupled fluid–solid-mechanics equation leading to asymmetric hydrodynamic resistances enabling a flow rectification of Newtonian fluids under the Stokes flow regime. Our theoretical model that captures the ceiling’s deformability and fluid’s compressibility^46^ is compared with the experimental results obtained from the shallow microfluidic device. A rectification ratio of up to 1.41 is experimentally demonstrated for both incompressible and compressible flows while a higher value is predicted for the more optimized channel geometry.

## Results and discussions

### Modeling

**Figure 1:**
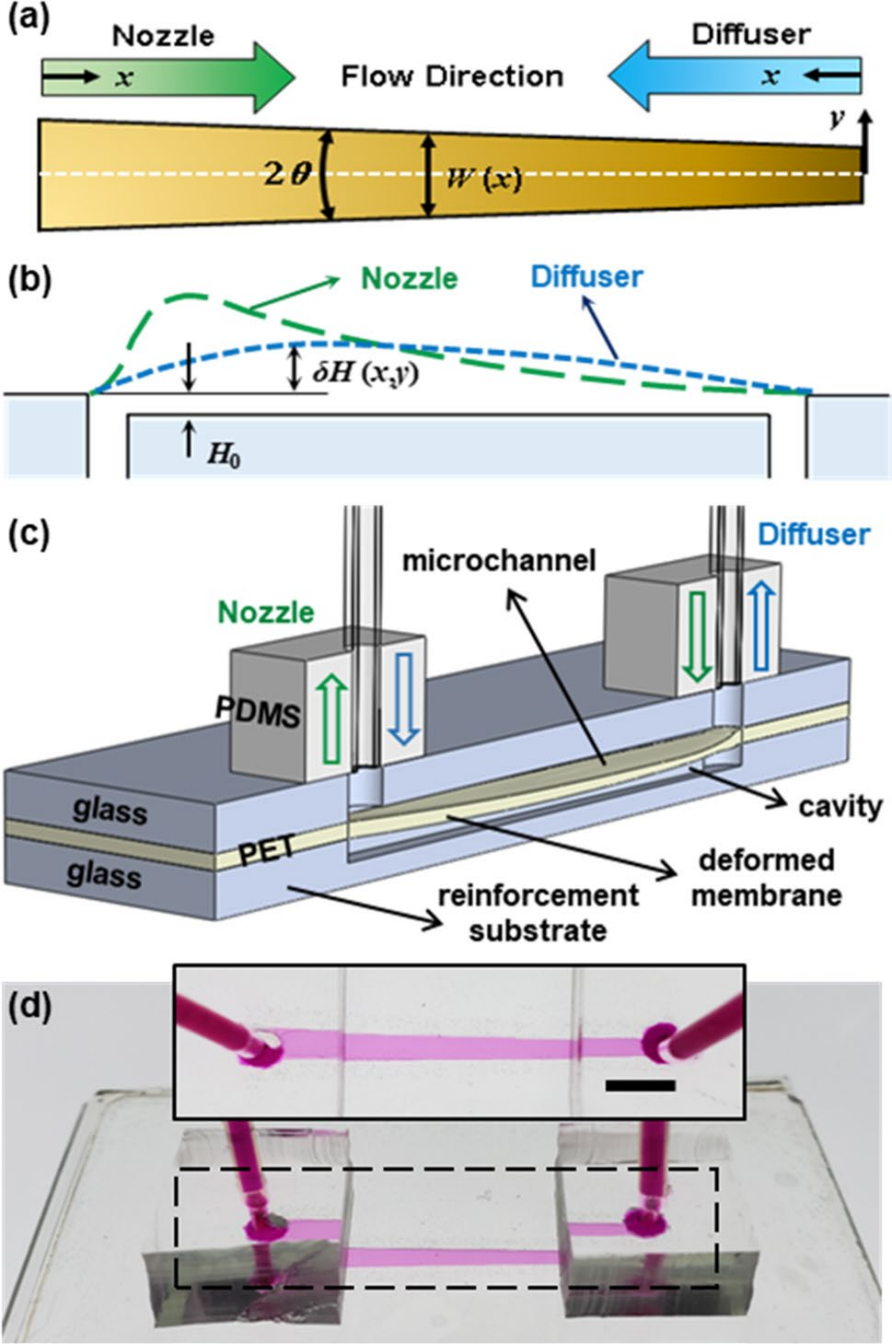
Schematic representations of a deformable nozzle/diffuser microchannel showing (**a**) a top-down view with the defined coordinate and channel dimensions, (**b**) a side view with the exemplary deformation profiles of a flexible ceiling under nozzle or diffuser configuration, (**c**) a 3D cross-sectional view with an assembled rectifier device; (**d**) a photograph of the fabricated deformable microchannel device ($W_{i} = 1{\text{ mm}}$, $W_{o} = 2{\text{ mm}}$, and $H_{0} = 10.9\,\upmu{\text{m}}$) filled with the dyed solution delineating the channel profile. Scale bar in the inset image is 2 mm.

Schematic representations of a fluid flow through a nozzle/diffuser microchannel with a deformable ceiling are shown in Fig. 1a,b. The ceiling membrane deflection depends on the fluid flow direction, *i.e.* nozzle versus diffuser, causing the overall hydrodynamic resistances of the diffuser and nozzle to differ from each other. The coupled fluid–solid mechanics model used in this work is based on the recent work by Christov et al.^44^, employing the lubrication theory, i.e. $H_{0} \ll W \ll L$*,* where $H_{0}$, $W$, and $L$ refer to the microchannel’s original height, width, and length, respectively. The thin-plate-bending framework is considered for the elastic deformation, which requires $\delta H \ll t \ll W$, where $\delta H$ is the membrane displacement and $t$ denotes the membrane thickness. Under these assumptions, the displacement of an arbitrary membrane’s infinitesimal slice across the channel can be correlated solely with the local fluid pressure within the channel^44^. The deflection profile of the slice is then obtained from the Euler–Bernoulli beam theory:

1 $$ \delta H\left( {x,\zeta } \right) = \frac{{p\left( x \right)W^{4} \left( x \right)}}{384D}f\left( \zeta \right), $$

where $\zeta \equiv 2y{/}W\left( x \right)$, $f\left( \zeta \right) = \left( {\zeta + 1} \right)^{2} \left( {\zeta - 1} \right)^{2}$, and $D = Et^{3} {/(}12\left( {1 - \nu^{2} } \right))$, in which $E$ and $\nu$ denote the membrane’s modulus of elasticity and Poisson’s ratio^47^. In addition, $p$ refers to the gauge pressure within the channel, which is constant at any x-plane (normal to the x-axis) according to the lubrication theory. For the detailed derivation of Eq. (1), see the reference^44^.

Since the membrane displacement is significantly smaller than the channel width and length, i.e. $\delta H \ll W \ll L$, the membrane slope is very small, i.e. $\partial \delta H{/}\partial x \ll 1$ and $\partial \delta H{/}\partial y \ll 1$. Under these situations and considering the sufficiently small nozzle/diffuser half-angle ($\theta$) with an attached flow throughout the channel, the local mass flow rate can be written as $\dot{m}\left( x \right) = - \frac{{dp\left( x \right){/}dx}}{12\mu }\rho \left( x \right)W\left( x \right)H^{3}_{\left( x \right)}$, where $\rho$ and $\mu$ refer to the fluid density and dynamic viscosity, respectively, and $H = H_{0} + \delta H$ shows the channel’s local height. In addition, the average of an arbitrary variable such as $\phi$ over a x-plane is obtained through $\phi_{\left( x \right)} = \frac{1}{2}\mathop \smallint \limits_{\zeta = - 1}^{\zeta = + 1} \phi \left( {x,\zeta } \right)d\zeta$. In a non-dimensional form, where the fluid density at standard pressure and temperature, $\rho_{{{\text{ref}}}}$, is used for the density unit, $L$ for the length unit, $\dot{m}_{{{\text{ref}}}}$ for the mass flow unit, $Q_{{{\text{ref}}}} = \dot{m}_{{{\text{ref}}}} {/}\rho_{{{\text{ref}}}}$ for the volumetric flow rate unit, and ${\Delta }p_{{{\text{ref}}}} = 12\mu LQ_{{{\text{ref}}}} {/}\left( {W_{i} H_{0}^{3} } \right)$ as the pressure unit, the dimensionless mass flow rate through the deformable channel, i.e. $\dot{m}^{*} \equiv \dot{m}{/}\dot{m}_{{{\text{ref}}}}$, can be written as

2 $$ \dot{m}^{*} = - \rho^{*} \frac{{dp^{*} \left( {x^{*} } \right)}}{{dx^{*} }}\frac{{W^{*} \left( {x^{*} } \right)}}{{W_{i}^{*} }}\frac{{H^{3}_{{\left( {x^{*} } \right)}} }}{{H_{0}^{3} }}, $$

where $W_{i}$ refers to the channel width at inlet, $\rho^{*} \equiv \rho {/}\rho_{{{\text{ref}}}}$, $p^{*} \equiv p{/}\Delta p_{{{\text{ref}}}}$, $x^{*} \equiv x{/}L$, $W^{*} \equiv W{/}L$, and $W_{i}^{*} \equiv W_{i} {/}L$. In general, the fluid density can be varied within the channel due to the compressibility effects^46^. For an isothermal fluid flow, we have $\rho^{*} = 1 + p\kappa_{T}$, where $\kappa_{T}$ denotes the isothermal compressibility of the fluid at standard pressure and temperature. From $H^{3} = H_{0}^{3} + 3H_{0}^{2} \delta H + 3H_{0} \left( {\delta H} \right)^{2} + \left( {\delta H} \right)^{3}$, we obtain

3 $$ \frac{{H^{3}_{{\left( {x^{*} } \right)}} }}{{H_{0}^{3} }} = 1 + 3\gamma \left( {x^{*} } \right)f\left( \zeta \right)_{{\left( {x^{*} } \right)}} + 3\gamma^{2} \left( {x^{*} } \right)f^{2} \left( \zeta \right)_{{\left( {x^{*} } \right)}} + \gamma^{3} \left( {x^{*} } \right)f^{3} \left( \zeta \right)_{{\left( {x^{*} } \right)}} , $$

where $\gamma \left( {x^{*} } \right) = p\left( {x^{*} } \right)W^{4} \left( {x^{*} } \right){/}\left( {384DH_{0} } \right)$, $f\left( \zeta \right) = 8{/}15$, $f^{2} \left( \zeta \right) = 128{/3}15$, and $f^{3} \left( \zeta \right) = 1024{/3003}$. The fluid is assumed to be discharged into ambient air at the outlet, i.e., $p^{*} = 0$ at $x^{*} = 1$. After defining $\xi \equiv 1 - x^{*}$ and substituting Eq. (3) into Eq. (2), we can derive an initial-value problem for an arbitrary mass flow rate of $\dot{m} = \dot{m}_{{{\text{ref}}}}$, i.e. $\dot{m}^{*} = 1$, as shown in the following first-order nonlinear ordinary differential equation (ODE):

4 $$ \begin{gathered}   \left( {1 + \alpha _{1} \tau ^{4} \left( \xi  \right)p^{*} \left( \xi  \right) + \alpha _{2} \tau ^{8} \left( \xi  \right)p^{{*2}} \left( \xi  \right) + \alpha _{3} \tau ^{{12}} \left( \xi  \right)p^{{*3}} \left( \xi  \right)} \right)\left( {1 + p^{*} \left( \xi  \right)\kappa _{T}^{*} } \right)\tau \left( \xi  \right)\frac{{dp^{*} \left( \xi  \right)}}{{d\xi }} = 1 \hfill \\   {\text{Initial value:  at}}\;\xi  = 0,\;p^{*}  = 0, \hfill \\  \end{gathered}  $$

where the dimensionless isothermal compressibility of the fluid is defined as $$\kappa_{T}^{*} \equiv \kappa_{T} \Delta p_{{{\text{ref}}}}$$. The width profile function $$\tau$$ is defined as $$\tau \left( \xi \right) \equiv W\left( \xi \right){/}W_{i}$$. For a nozzle/diffuer microchannel with a linear variation of the width, $$\tau \left( \xi \right) = \tau_{0} + \left( {1 - \tau_{0} } \right)\xi$$, where $$\tau_{0} \equiv W_{0} {/}W_{i}$$ and $$W_{o}$$ refers to the channel width at outlet. For the special case of $$\tau = 1$$, the governing equation is reduced to that of the fluid flow through a deformable shallow straight microchannel^45^. The three coefficients of $$\alpha_{i}$$ are $$\alpha_{1} = \left( {8{/}5} \right)\chi$$, $$\alpha_{2} = \left( {128{/}105} \right)\chi^{2}$$, and $$\alpha_{3} = \left( {1024{/}3003} \right)\chi^{3}$$, where $$\chi$$ is denoted as a flexibility parameter, that is, $$\chi \equiv \Delta p_{{{\text{ref}}}} 
W_{i}^{4} {/}\left( {384DH_{0} } \right).$$ It has been previously shown that $$\chi$$ is a crucial dimensionless parameter in the analyses of a fluid flow within deformable microchannels and scales linearly with applied pressure difference $$\Delta p$$ for the given microchannel dimensions and membrane properties^44,45^. Equation (4) is solved numerically to find the pressure distribution within the channel, i.e., $$p^{*} \left( \xi \right)$$, and other fluid–solid characteristics. In case of an incompressible flow, $$\rho$$ (or $$\rho^{*}$$) remains constant, and thus Eq. (2) can be rewritten for the dimensionless volumetric flow rate, $$Q^{*} \equiv Q{/}Q_{{{\text{ref}}}} = - \left( {dp^{*} \left( {x^{*} } \right){/}dx^{*} } \right)\left( {W^{*} \left( {x^{*} } \right){/}W_{i}^{*} } \right)\left( {H^{3}_{{\left( {x^{*} } \right)}} {/}H_{0}^{3} } \right)$$.

### Experimental validation

To validate the proposed coupled fluid–solid mechanics model, we fabricated asymmetrically-shaped (e.g., nozzle/diffuser) deformable microchannels with ultralow height-to-width aspect ratios. Figure 1c shows a schematic diagram of the deformable microchannel device consisting of a polyethylene terephthalate (PET) membrane sandwiched between two pre-etched glass slides and polydimethylsiloxane (PDMS) interconnects. An etched trench in the top glass slide serves as a shallow microchannel while an etched cavity in the bottom slide provides a room for the membrane deflection. The bottom slide is needed to reinforce the bonding strength for high-$\Delta p$ operations. A photograph of the fabricated device is shown in Fig. 1d where the shallow microchannel was visualized with a dye solution. The detailed fabrication procedure has been reported elsewhere^48^. Among several width profiles that have been theoretically studied, we chose 1 mm and 2 mm for the small and large widths of the fabricated nozzle/diffuser channels, respectively (see Supplemental Materials A for more details). With $L = 22.9\;{\text{mm}}$, the half angle, $\theta$, is 1.25 degree, which is sufficiently small for the Poiseuille flow characteristics of a straight channel and the Euler–Bernoulli beam theory to be locally valid.

**Figure 2:**
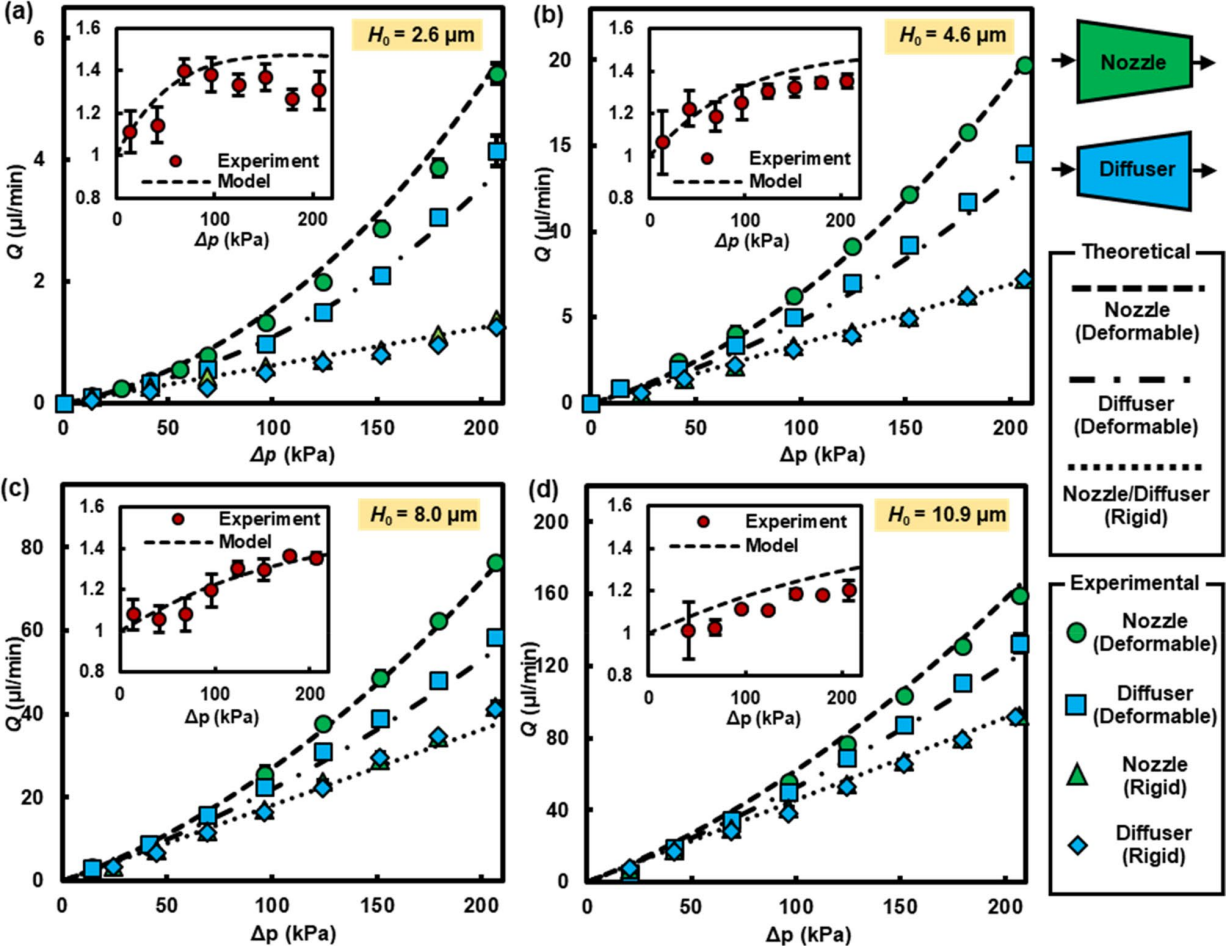
Volumetric flow rates, $Q$, of DI water as a function of pressure difference, $\Delta p$, across deformable (or flexible) and rigid nozzle/diffuser microchannels (with the widths of 1 and 2 mm and length of 22.9 mm) for various original channel heights of $H_{0} =$ (**a**) $2.6 \pm 0.2\,\upmu{\text{m}}$, (**b**) $4.6 \pm 0.2\,\upmu{\text{m}}$, (**c**) $8.0 \pm 0.3\,\upmu{\text{m}}$, and (**d**) 10$.9 \pm 0.3\,\upmu{\text{m}}$. The dashed, dash-dotted, and dotted lines show the results obtained from the fluid–solid coupled model for deformable nozzle, deformable diffuser, and rigid nozzle/diffuser, respectively. The inset graphs show the rectification ratio, $\eta$, as a function of $\Delta p$ for each deformable nozzle/diffuser microchannel. The error bars were averaged over three runs and may not be visible if they are smaller than the marker’s size.

Theoretical and experimental results of the volumetric flow rates over the applied pressure difference of 14–206 kPa were obtained for DI water flows through deformable/rigid nozzle/diffuser microchannels with four different original channel heights (2.6, 4.6, 8.0, and 10.9 μm) and are shown in Fig. 2a–d. The theoretical results of flexible and rigid channels were derived from Eq. (4) and Eq. S(d), respectively. Because of the linear nature of equations governing the low-Reynolds-number flows, rigid nozzle and diffuser microchannels exhibit the linear relationship between $\Delta p$ and $Q$ and generate the identical hydrodynamic resistance, i.e., no net flow rectification. However, regardless of the original channel height, deformable nozzle/diffuser channels display superlinear $\Delta p$ vs. $Q$ characteristics, in which a deformable nozzle delivers a larger volumetric flow rate compared to a deformable diffuser under the same $\Delta p$, resulting in a flow rectification. Figure S2 in Supplementary Materials displays the average membrane displacements, $\delta H$, calculated along the channel direction for both nozzle and diffuser configurations and suggests that an effectively larger $\delta H$ in a nozzle leads to a reduction in net hydrodynamic resistance and a greater flow rate in comparison to that of a diffuser. Note that the calculated Reynolds number in the mid-plane ($x^{*} = 0.5$) varies from $O\left( {10^{ - 3} } \right)$ to $O\left( {10^{0} } \right)$ (see Fig. S3 in Supplementary Materials), verifying that rectification of a low-Reynolds–number flow can be realized for Newtonian fluids.

A key performance metric of a fluidic rectifier is a rectification ratio, $\eta$, defined as a ratio of a mass (or volumetric) flow rate through nozzle to that of diffuser under the same $\Delta p$, i.e., $\eta \equiv \dot{m}_{{{\text{Nozzle}}}} {/}\dot{m}_{{{\text{Diffuser}}}}$ (or $Q_{{{\text{Nozzle}}}} {/}Q_{{{\text{Diffuser}}}}$). $\eta$ is plotted in each inset of Fig. 2 as a function of $\Delta p$ for four microchannels with different $H_{0}$. The largest $\eta$ of 1.41 was attained for the microchannel with $H_{0} = 2.6\,\upmu{\text{m}}$. Both theoretical and experimental results show that $\eta$ increases with increasing $\Delta p$ in small $\Delta p$ but plateaus at higher $\Delta p$. The limiting behaviors of $\eta$ under extremely small or large $\Delta p$ are theoretically studied in Supplementary Materials C, which concludes that $\eta$ approaches to 1 and no rectification is generated for both limiting cases of $\Delta p$. Meaningful rectification ($\eta > 1$) or the maximum $\eta$ value can be achieved at intermediate $\Delta p$. The flexibility parameter, $\chi$, captures how $\eta$ can be affected by $\Delta p$ and other parameters such as the channel geometries and membrane properties. For example, very small $\Delta p$ means $\chi \ll 1$, diminishing the role of the nonlinear terms in Eq. (4) and making deformable microchannels exhibit a linear characteristic behavior similar to their rigid counterparts. Conversely, when high $\Delta p$ is applied ($\chi \gg 1$), the highest-order term ($\chi^{3}$) becomes dominant in Eq. (4). If an incompressible flow is assumed, differences in membrane deflection for nozzle vs. diffuser configurations become negligible and the microchannel behaves more like a straight deformable channel, i.e., no rectification. As shown in our earlier work^45^, all of the nonlinear terms, $\alpha_{i} \tau^{4i} p^{*i} \left( {i = 1, 2, 3} \right)$, become activated in Eq. (4) for intermediate $\Delta p$ or $10^{ - 1} < \chi < 10^{1}$, in which the embedded nonlinear effect induces directional dependence on flow resistance and in turn produces flow rectification. This implies that a meaningful rectification of Stokes flows is attainable but not guaranteed for Newtonian fluids in asymmetrically-shaped deformable microchannels and that the operating pressure range should be determined for the optimal $\chi$ for given channel geometries.

**Figure 3:**
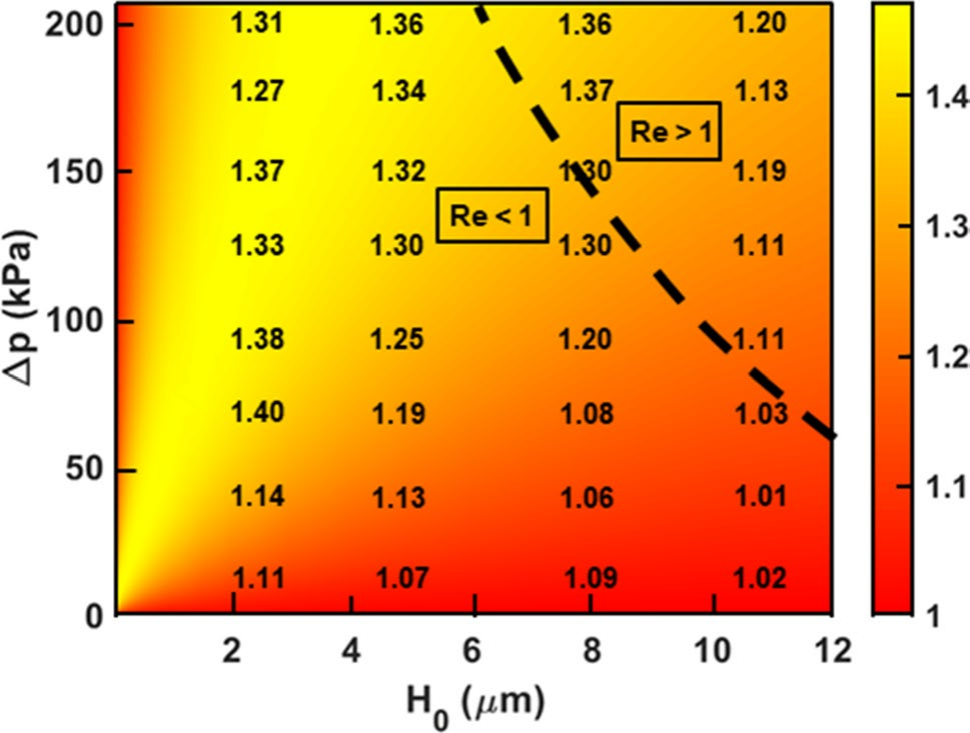
The rectification ratio ($\eta$) contour plot obtained from the coupled fluid–solid-mechanics model for a DI water flow through the nozzle/diffuser microchannels with the widths of 1 mm and 2 mm. The tabulated values are the experimental results noted from Fig. 2 at the corresponding ($H_{0}$, $\Delta p$). The dotted line indicates the conditions where the averaged Reynolds number at the midplane ($x^{*} = 0.5$) becomes 1.

It can be also observed in Fig. 2 that an increase in $H_{0}$ attenuates the resulting $\eta$ in the given $\Delta p$ range. As $H_{0}$ becomes greater, a relative contribution of the membrane deformation ($\delta H$) to the overall channel height ($H_{0} + \Delta H$) is diminished, and consequently a deformation-induced change in the hydrodynamic resistance plays a less noticeable role in net hydrodynamic resistance. See Fig. S4a,b in Supplementary Materials for plots of relative membrane displacements at nozzle/diffuser inlets and averaged hydrodynamic resistances for various $H_{0} .$ Discrepancies between the averaged hydrodynamic resistances of the nozzle and diffuser are the largest for $H_{0} = 2.6\,\upmu{\text{m}}$ and decrease with increasing $H_{0}$. The declining trend of $\eta$ for increasing $H_{0}$ was further verified in Fig. S5 where the maximum rectification ratio was droped to around 1.1 for the deformable nozzle/diffuser microchannel with $H_{0} = 41\,\upmu{\text{m}}$. The dependence of $H_{0}$ and $\Delta p$ on $\eta$ is summarized in a contour plot of Fig. 3 in which the bright yellow indicates the highest $\eta$ region and the red the lowest $\eta$ region. The contour plot was obtained from the proposed fluid–solid model and compared with the experimental values noted from Fig. 2. The model predicts that a range of $\Delta p$ at which the maximum $\eta$ can be attained increases with $H_{0}$; for instance, $\eta_{{{\text{max}}}}$ ~ 1.47 is achieved around $\Delta p$ = 62, 117, and 200 kPa for the microchannels with $H_{0} = 1{ }, 2, \,{\text{and}}\, 4\,\upmu{\text{m}}$, respectively. It is anticipated that $\eta_{{{\text{max}}}}$ can be reached at higher $\Delta p$ for microchannels with large $H_{0}$ (not shown in Fig. 3), but we need to note that a microchannel with large $H_{0}$ and/or operating under high $\Delta p$ make the proposed model deviate from the Stokes flow assumption (*Re* > 1 in the upper right corner of the contour plot).

Strictly speaking, the flexible ceiling of the proposed device can be considered as a moving part, and one could argue that the device suffers from the same issues (i.e., clogging due to the structural adhesion, interference with biospecies, etc.) encountered by the microfluidic diodes with the moving components. However, these issues are not problematic for the proposed device because the deflection of the flexible ceiling occurs in the direction of increasing the hydrodynamic conductance (i.e., the ceiling is moving away from the bottom wall enlarging the channel’s cross-sectional area), which is opposite to the working principle of the microfluidic diodes.

### Flow rectification of other Newtonian fluids

**Figure 4:**
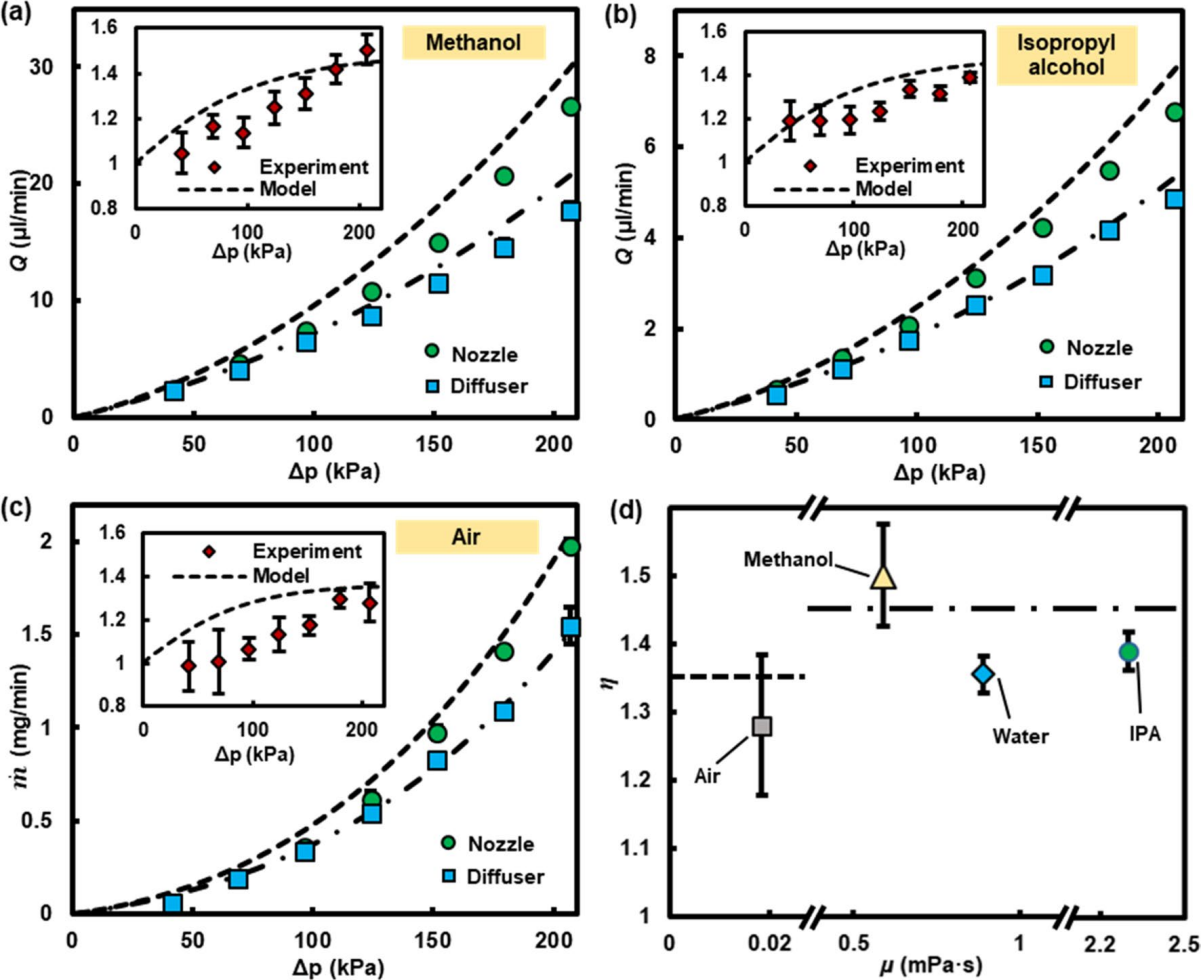
Volumetric flow rates, *Q*, of (**a**) methanol and (**b**) isopropyl alcohol as a function of pressure difference, $\Delta p$, across the deformable nozzle/diffuser microchannel ($W_{i,o} = 1 \,{\text{or}}\, 2\,{\text{mm}}$, $H_{0} = 4.6\,\upmu{\text{m}}$). (**c**) Mass flow rates, $\dot{m}$, of air as a function of $\Delta p$ for the same microchannel. The dashed and dashed-dotted lines indicate the results obtained from the fluid–solid-mechanics model for the deformable nozzle and diffuser, respectively. The insets show the rectification ratio, $\eta$, as a function of $\Delta p$. (**d**) $\eta$ plotted as the fluid’s viscosity. The dashed and dashed-dotted lines represent the theoretical model for compressible and incompressible fluids, respectively.

Rectification behaviors of the asymmetrically-shaped shallow deformable microchannels were evaluated for Stokes flows of other Newtonian fluids. We chose methanol ($\mu = 5.9 \times 10^{ - 4} {\text{Pa s}}$) and isopropyl alcohol (IPA, $\mu = 2.3 \times 10^{ - 3} {\text{Pa s}}$) as working fluids because their viscosities are meaningfully different from that of water ($\mu = 8.9 \times 10^{ - 4} {\text{Pa s}}$). Figure 4a,b show the theoretical and experimental results of *Q* vs. $\Delta p$ for methanol and IPA, respectively, across the deformable nozzle/diffuser microchannel with $H_{0} = 4.6\,\upmu{\text{m}}$. *Q* is largest for methanol followed by water and IPA for the same $\Delta p$ because a flow rate inversely scales with the fluid’s viscosity. The theoretical rectification ratio represented by the dotted line in the inset graphs, however, remains constant according to the proposed model of Eq. (4) (also see Fig. 4d). The dimensionless flow rate and consequently the rectification ratio depend on the pressure distribution within the microchannel, which is solely dictated by the membrane properties as well as the channel geometry but not by the fluid’s viscosity.

Equation (4) captures both channel’s deformability and fluid compressibility, leading to the prediction of a universal rectifier for Stokes flows of both liquids and gases. Figure 4c shows the theoretical and experimental results of $\dot{m}$ vs. $\Delta p$ for air ($\mu = 1.8 \times 10^{ - 5} {\text{Pa s}}$) across the same device ($H_{0} = 4.6\,\upmu{\text{m}}$) with $\eta$ in the inset. The results reveal that a nozzle delivers a larger $\dot{m}$ of air than a diffuser and confirm that the idea of Stokes flow rectification through shallow deformable microchannels can be extended to compressible fluids. It it important to note that the neglect of the fluid compressibility underestimates $\dot{m}$ especially for the high $\Delta p$ range and the fluid compressibility must be considered to accurately predict $\dot{m}$ of a compressible flow^46^. In the Supplementary Material E, a systematic study is presented to reveal the effect of the fluid compressibility and channel deformability on flow characteristics across both rigid and deformable microchannels. We found that $\eta$ of the air flow is somewhat smaller than that of the liquid flows (see Fig. 4d). For a compressible flow, the fluid density decreases towards the downstream of the channel as the pressure decreases. Such a decaying density variation causes the fluid speed, shear rate, and net fluidic resistance to increase along the channel. Consequently, the relative contribution of the nozzle’s wide portion in upstream to the overall hydrodynamic resistance decreases, undercutting the effect of displacement-induced change in hydrodynamic resistance. The opposite is true for the diffuser because the wider portion of the diffuser is in downstream. The net effect is a reduction in $\eta$. Despite the lower $\eta$ of compressible flows, our work reports the first demonstration of rectifying an equilibrium gas flow (Knudsen number smaller than 0.001) under the Stokes flow regime with the maximum $\eta$ ~ 1.39.

### Optimizing rectification performance

**Figure 5:**
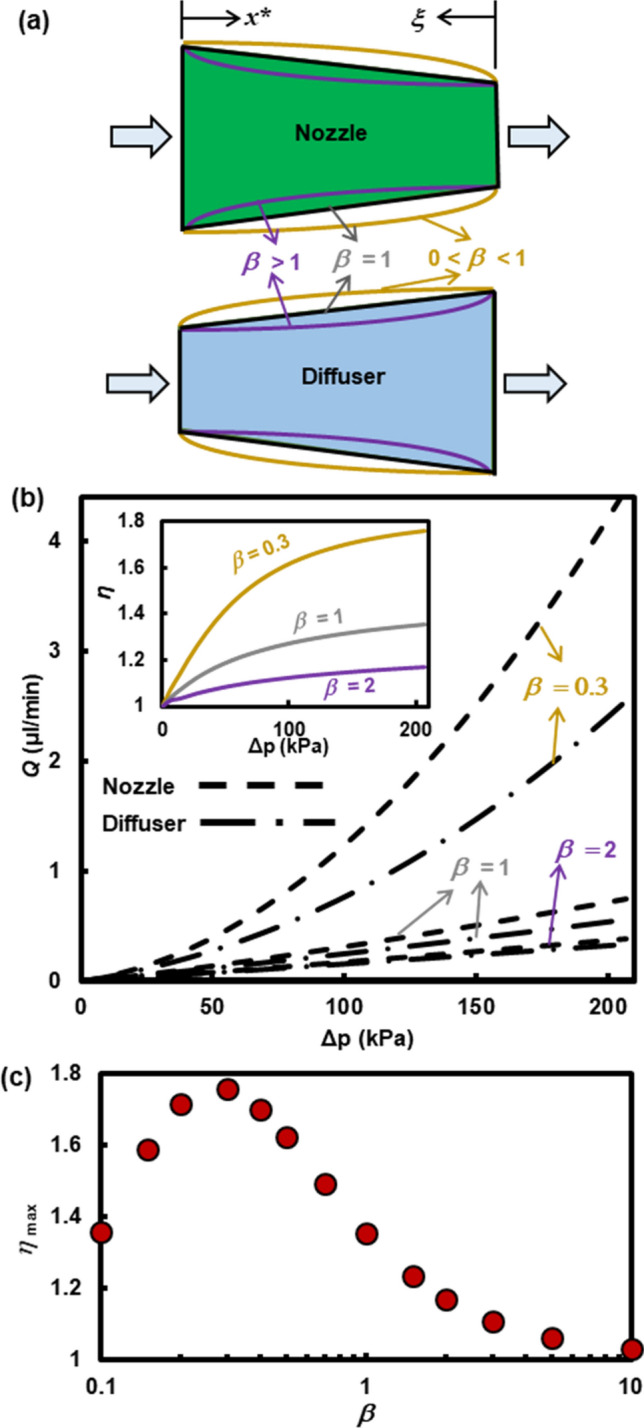
(**a**) Schematic diagrams of the nonlinear width profiles characterized by a parameter $\beta$ for the nozzle direction, $W_{N} = W_{o} + \left( {W_{i} - W_{o} } \right)\xi^{\beta }$, and the diffuser direction, $W_{D} = W_{i} + \left( {W_{o} - W_{i} } \right)\left( {1 - \xi } \right)^{\beta }$. (**b**) Volumetric flow rates, *Q*, of water calculated from the fluid–solid-mechanics model as a function of pressure difference, $\Delta p$, across the deformable nozzle/diffuser microchannels ($W_{{{\text{small}}}} = 0.3\,{\text{mm}}$, $W_{{{\text{large}}}} = 2\,{\text{mm}}$, $H_{0} = 2.6\,\upmu{\text{m}}$, and $\theta = 1.25^\circ$) with three different $\beta$ values (0.3, 1, and 2). The inset shows the rectification ratio, $\eta$, as a function of $\Delta p$. (**c**) The maximum $\eta$ for the microchannels as a function of the nonlinear sidewall parameter $\beta$ over $\Delta p$ of 14–206 kPa.

Passive rectifiers with improved performances can be realized through optimizing the width profiles. From the schematic representation of the nonlinear width profiles in Fig. 5a, we can express the nonlinear width profile function, $\tau_{N} \left( \xi \right) = W_{N} {/}W_{i,N} = \tau_{o,N} + \left( {1 - \tau_{o,N} } \right)\xi^{\beta }$, for the nozzle direction and, $\tau_{D} \left( \xi \right) = W_{D} {/}W_{i,D} = 1 + \left( {\tau_{o,D} - 1} \right)\left( {1 - \xi } \right)^{\beta }$, for the diffuser direction, where $\tau_{o,N} = W_{o,N} {/}W_{i,N}$, $\tau_{o,D} = W_{o,D} {/}W_{i,D}$, and $\beta$ is an arbitrary variable for adjusting the width profile. Here, we select the nominal dimension for deformable nozzle/diffuser microchannels to be $W_{{{\text{small}}}} = 0.3\,{\text{mm}}$, $W_{{{\text{large}}}} = 2\,{\text{mm}}$, $H_{0} = 2.6\,\upmu{\text{m}}$, and $\theta = 1.25^\circ$. Based on the calculations of the proposed fluid–solid-mechanics model, we plot *Q* vs. $\Delta p$ of water flow in Fig. 5b for three different $\beta$ values (0.3, 1, and 2). The microchannel with the width profile of $\beta = 0.3$ delivers a larger *Q* than the other two because its larger averaged cross-section area lowers the hydrodynamic resistance.

More interestingly, we observe in the inset of Fig. 5b that the microchannel with the width profile of $\beta = 0.3$ exhibits a larger $\eta$ than that of the linear profile ($\beta = 1$) while the microchannel with the width profile of $\beta = 2$ results in a smaller $\eta$. This observation is consistent with the working principle of the proposed rectifiers. In the nozzle configuration, the wide portion of the flow entrance becomes extended in the $\beta = 0.3$ case and is subject to the high pressure side, inducing a larger membrane deflection $\delta H$. When the flow direction is reversed, i.e., in the diffuser configuration, the extended portion is in downstream and experiences the lower fluid pressure lessening $\delta H$. This causes a greater difference in the hydrodynamic resistance, which in turn increaes $\eta$. Figure 5c presents the maximum $\eta$ for the microchannel with different width profile functions when operated with $\Delta p$ of 14–206 kPa. As explained above, extending the wide portion of the channel by decreasing $\beta$ increases $\eta$ until $\beta$ becomes 0.3. For $\beta < 0.3$, $\eta$ also decreases as $\beta$ gets smaller, since too much extension of the wide portion of the channel leads to the large $\delta H$ for the diffuser direction as well, reducing the contrast between the nozzle and diffuser characteristics. The maximum $\eta$ is calculated to be 1.76 for the channel with $\beta = 0.3$. It should be noted that this value is specific for the dimension of the selected asymmetric microchannel and a larger $\eta$ could be achieved using different channel dimensions and more optimized width profile functions.

## Conclusion

Passive rectification of the Stokes flow has been limited to non-Newtonian fluids or, for Newtonian fluids, entails mechanical intervention that may cause channel blockage and interfere the analyte transport. In this paper we experimentally demonstrated that a rectification ratio of $\eta$ ~ 1.41 was achieved for Newtonian fluid flows with the Reynolds number well below 1. A source of nonlinearity necessary for flow rectification stems from the coupled fluid–solid mechanics in an asymmetrical-shaped, ultralow-aspect-ratio microchannel with deformable ceiling. The analytical model derived here reasonably captures the nonlinear behaviors of direction-dependent hydrodynamic resistance and allows us to reliably design a microchannel for better rectifying performance ($\eta$ ~ 1.8). This rectifying scheme can be universally applied for both incompressible and compressible Newtonian fluids. The rectification ratios obtained from the Stokes flows of water, methanol, isopropyl alcohol and dry air were similar in magnitude, demonstrating that the rectifier's performance is independent of the fluid viscosity. The proposed shallow deformable microfluidic platform along with the analytical modeling technique can be exploited to develop micropumps and microvalves with precise flow-rate control for potential bioapplications such as selective species transport and drug delivery.

## Materials and methods

### Materials

Corning plain microscope slides (75 × 50 mm size, 1 mm thickness) were purchased from Fisher Scientific and thoroughly cleaned using piranha etch (a mixture of sulfuric acid, hydrogen peroxide and water). Polyethylene terephthalate (PET) films ($t = 100 \pm 5\,\upmu{\text{m}}$, $E = 2{-}4{\text{ GPa}}$, and $\nu \approx 0.4$) were purchased from GoodFellow and thoroughly cleaned using a standard degrease process. SU-8 2100 and SU-8 2000 thinner were purchased from Microchem Corp. and mixed to result in desired film thicknesses. Photoresist AZ4620 and Developer AZ 400 K were purchased from Integrated Micro Materials. Buffered oxide etchant (BOE; HF(48%):NH_4_F(40%):HCl(37%) = 1:6:1.4 by vol.) was purchased from MicroChemicals GmbH. Sylgard 184 silicone elastomer was purchased from Dow Corning for polydimethylsiloxane (PDMS) fluidic interconnects.

### Device design and fabrication

An asymmetrically-shaped (e.g., nozzle/diffuser) deformable microchannel device is schematically shown in Fig. 1c. The fabrication of the proposed deformable shallow microchannels is similar to the procedures previously reported^48^ and briefly described here. The device consists of two pre-etched microscope slides sandwiching a 100-μm-thick PET film. One microscope slide was patterned using photolithography, etched using the BOE solution for shallow trenches (various channel depths of 2.6, 4.6, 8.0, and 10.9 μm), and drilled to create two via holes for inlet and outlet. The other microscope slide was also patterned using the same mask but this time etched much deeper (> 45 μm) allowing the PET membrane to freely deform under pressure. Prior to bonding the glass slide with the shallow channel and the membrane, a 2-μm-thick SU-8 layer was coated onto the PET film for adhesive bonding. The glass slide with the deeper trench serves as bonding reinforcement and was bonded to the bare side of the membrane using a 16-μm-coated SU8 layer ($E \sim { }2{\text{ GPa}}$)^48^. A blank glass slide was used to make a rigid microchannel counterpart for comparison with each deformable channel. The structural effects of the adhesive layers can be taken into account in our model by using the transformed-section method^45^. The equivalent membrane thickness is calculated from $\tilde{t} = \sqrt[3]{{12I/W}}$, where $I$ denotes the SU8-PET-SU8 membrane’s moment of inertia. The equivalent membrane thickness, i.e., $\tilde{t}$ replaces $t$ in the parameter $D$ of the flexibility parameter. Considering the aforementioned values for thickness and modulus of elasticity of different layers, we obtain $\tilde{t} \approx 113\,\upmu{\text{m}}$.

### Flow rate measurements

A pressure-driven flow of liquids (e.g., water, isopropyl alcohol, and ethanol) was generated at constant pressure levels by employing a compressed air to push a working fluid out of a custom-made flask with two ports. A pressure source is connected to one end of the flask via a pressure regulator (PneumaticPlus, PPR2-N02BG-4 Miniature Air Pressure Regulator), while the other end is connected to the microchannel’s inlet. The liquid discharged from the microchannel was guided through a glass capillary tube with a known inner diameter, where the meniscus position was monitored over time to calculate the volumetric flow rate^48^. Air was chosen as a model compressible fluid. A small water plug was placed within the outlet tube and pushed out by the applied air pressure. The movement of the water plug was monitored with a camera attached to a stereomicroscope, and the linear velocity of liquid meniscus was measured to calculate the volumetric flow rate of air and consequently the mass flow rate through the channel^46^.

## Supplementary Information

Supplementary Information.
