# Supplementary material for: A passive Stokes flow rectifier for Newtonian fluids
Source: Sci Rep. 2021 May 13;11:10182. doi: 10.1038/s41598-021-89699-y (PMC8119468; doi:10.1038/s41598-021-89699-y)
Supplement: Supplementary file 1 — Supplementary Information. [file 41598_2021_89699_MOESM1_ESM.pdf]

Supplementary Information

Aryan Mehboudi[1] and Junghoon Yeom[2,*]

1. Department of Mechanical Engineering, The University of Texas, Austin, TX 78758 USA
2. Code 6354, Multifunctional Materials Branch, Materials Science and Technology Division, Naval Research Laboratory, Washington, DC 20375 USA

* junghoon.yeom@nrl.navy.mil

A. Microchannel design

The fluid-solid characteristics of shallow deformable microchannels rely on the following three general categories: 1) microchannel cross-sectional dimensions: W_i, W_o, and H_0, 2) membrane thickness and structural properties, which are lumped into D, and 3) pressure difference across the microchannel, Δp. Once the channels are fabricated, the first two categories become fixed, and Δp dictates the channel characteristic behaviors. From our previous study, a 100-micron-thick PET film proved to be the optimal flexible ceiling because the resulting D parameter allows the membrane to be sufficiently displaced with the reasonable Δp range. The question still remains what microchannel cross-sectional dimensions should be used for experimental demonstration. We have theoretically studied the performance of numerous fluidic rectifiers that have different W_i and W_o for a given original height, $H_0 = 2.6\ \mu m$ and a channel length, $L = 22.9$ mm. Under the experimentally accessible range of applied pressure difference (14 kPa $< \Delta p <$ 206 kPa), the volumetric flow rates under nozzle and diffuser configurations and the corresponding rectification ratios (η) for some representative microchannels are plotted in Fig. S1. The nozzle/diffuser microchannel with 1 and 2 mm of small and large widths is chosen for experimental demonstration thanks to its relatively large rectification ratio. Note that the half-angle (θ) is not a design parameter, since it needs to be very small ($\theta \approx 0$) so that the Poiseuille flow characteristics for a straight channel and the Euler-Bernoulli beam theory are locally valid. In our study, $\theta = \arctan\big((W_o - W_i)/2/L\big) \approx 0.022$ rad $= 1.25°$, reasonably meeting the aforementioned requirement.

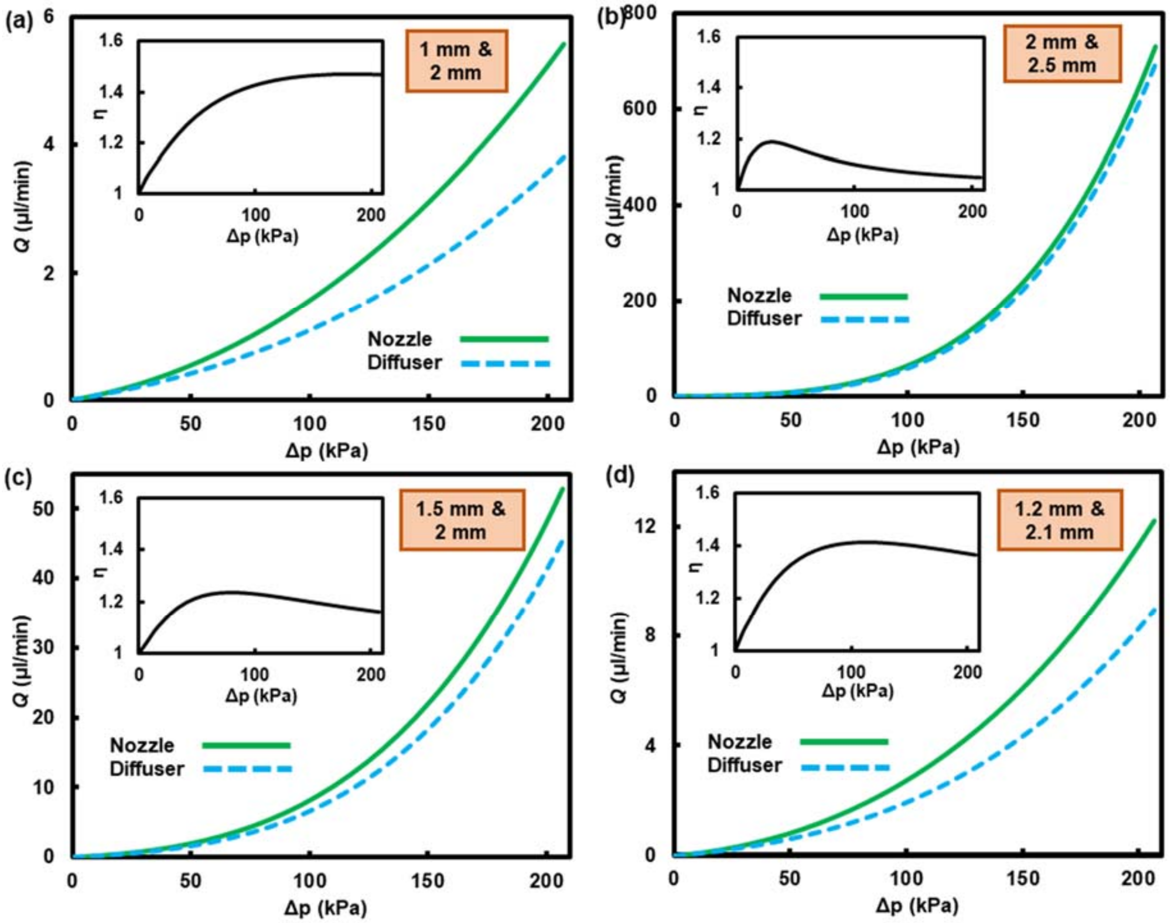

Figure S1. Volumetric flow rates (Q) of water obtained from the 1-D coupled fluid-solid-mechanics model as a function of pressure difference across the deformable nozzle/diffuser microchannels of $H_0 = 2.6\ \mu m$ in height with the half-angle of $\theta = 1.25°$ for various small and large widths: (a) 1 mm and 2 mm, (b) 2 mm and 2.5 mm, (c) 1.5 mm and 2 mm, and (d) 1.2 mm and 2.1 mm. The inset images show the rectification ratio, *i.e.*, $\eta = Q_{\text{Nozzle}}/Q_{\text{Diffuser}}$, as a function of pressure difference.

B. Membrane displacements and Reynolds number calculations in microchannels

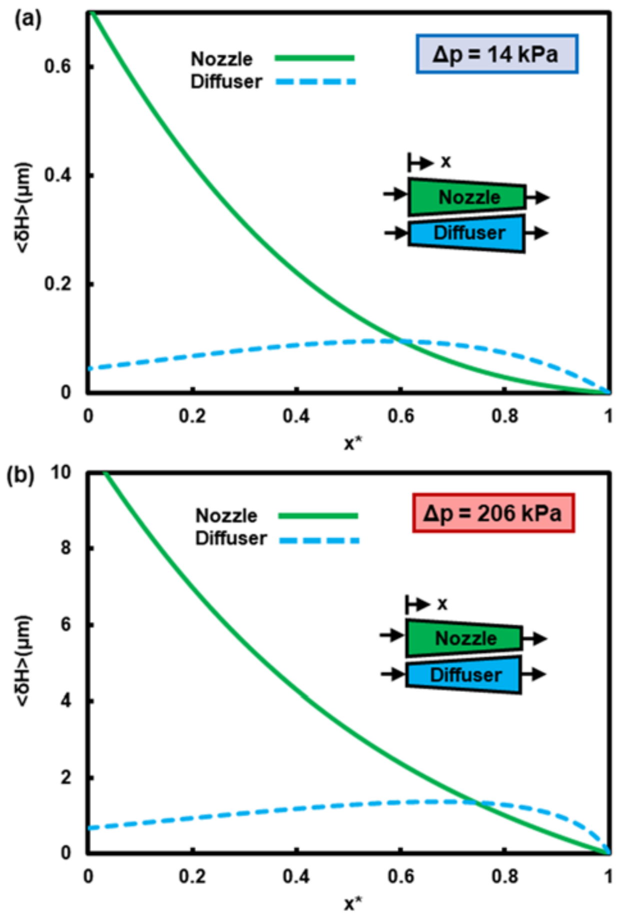

Figure S2. The averaged membrane displacements $\langle \delta H \rangle$ at inlet are evaluted from the proposed model for the deformable nozzle/diffuser microchannel (widths = 1 and 2 mm, length = 22.9 mm, height = 2.6 μm) under (a) Δp = 14 kPa and (b) Δp = 206 kPa.

Note that the boundary effects at inlet and outlet ($x^* = 0$ and 1) are not factored in the model and the membrane near the boundary is anchored onto the substrate in real devices. This means that δH of the nozzel should be close to 0 at inlet and therefore actual differences in $\langle \delta H \rangle$ for nozzle vs. diffuser are not as significant as the calculated profiles suggest.

The Reynolds number is calculated for four different nozzle/diffuser microchannels using $Re = \rho \bar{u} D_h / \mu$, in which $\bar{u}$ and D_h denote the average velocity of the flow-wise component and the hydraulic diameter of the microchannel, respectively. Here For shallow microchannels, i.e., $H_0 \ll W$, we consider the hydraulic diameter as $D_h(x) \approx 2(H_0 + \langle \delta H \rangle_{(x)})$. We can write $\bar{u} = \frac{Q}{W(x)(H_0 + \langle \delta H \rangle_{(x)})} = \frac{2Q}{W(x) D_h(x)}$. From these relations, we can compute the Reynolds number by using $\mathrm{Re}(x) = 2\rho Q / \mu W(x)$. Q is obtained from the coupled fluid-solid mechanics model (Eq. 4). Since W varies along the channel length, the Reynolds number is evaluated at the mid-plane ($x^* = 0.5$) and shown in Fig. S3. From Fig. S3, we observe that the flow condition of the microchannels with H_0 = 2.6 μm and 4.6 μm falls under the Stokes flow regime ($\mathrm{Re} < 1$) for all investigated Δp range and

that the flow condition of the microchannels with $H_0 = 8.0$ µm and 10.9 µm satifies Re < 1 for the majority of the Δp range.

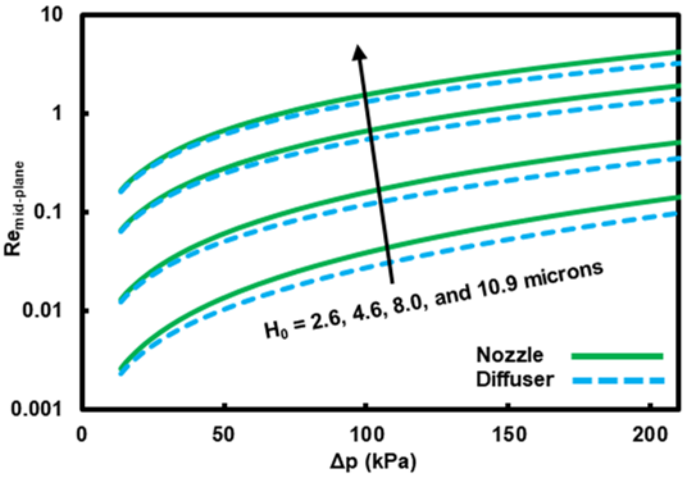

Figure S3. Reynolds number computed based on the channel width at the midplane ($x^* = 0.5$) for water flow through deformable nozzle/diffuser microchannels with the half-angle of $\theta = 1.25°$, small and large widths of 1 mm and 2 mm, and various original channel heights.

C. Limiting behaviors of the analytical model

The key dimensionless parameter in the proposed model and the shallow deformable microfluidics in general is the flexibility parameter, $\chi = \Delta p_{\text{ref}} W_i^4/(384 D H_0)$. A small value of χ indicates that the microchannel behaves like a rigid one while a large value means the microchannel with the highly flexible ceiling. From the variables that makes up χ, one can attain the flow characteristics of a highly deformable microchannel when (i) the applied pressure (Δp_{ref}) is high, (ii) the channel width (W_i) is large, and (iii) the fleixible ceiling is highly compliant (i.e., small D). The effect of the original channel height (H_0) on a flow in the deformable channel may not be obvious, but the smaller H_0, the more significant the relative volume change caused by the movable ceiling becomes. Below we mathematically show that the rectification effect disappears for both extreme cases: χ being very small or very large.

C.1 Extremely small flexibility parameter ($\chi \ll 1$)

For a rigid channel, the flexibility parameter equals zero, *i.e.*, $\chi = 0$. For deformable channels under sufficiently small pressure differences, the flexibility parameter can also be very small, *i.e.* $\chi \approx 0$, resembling the behavior of the rigid channel. Under these conditions, the terms with $\alpha_i : i = 1$, 2, and 3 multipliers vanish from the coupled fluid-solid-mechanics governing equation presented in Eq. 4. The simplified ODE is

$$\tau(\xi)(1 + p^*(\xi)\kappa_T^*)\frac{dp^*(\xi)}{d\xi} = 1 \tag{a}$$

Initial value: at $\xi = 0$, $p^* = 0$.

For a nozzle/diffuser, where $\tau(\xi) = \tau_0 + (1 - \tau_0)\xi$, one can solve Eq. (B-1) to obtain the following analytical expression for the pressure distribution within the channel:

$$p^*(\xi) = \frac{1}{\kappa_T^*}\left(\sqrt{1 + \frac{2\kappa_T^*}{1 - \tau_0}\ln\frac{\tau(\xi)}{\tau_0}} - 1\right). \tag{b}$$

The dimensionless pressure at inlet can then be obtained as

$$p_i^* = \frac{1}{\kappa_T^*}\left(\sqrt{1 + 2\kappa_T^*\frac{\ln\tau_0}{\tau_0 - 1}} - 1\right), \tag{c}$$

Since $\Delta p = p_i^* \Delta p_{\text{ref}}$, $\Delta p_{\text{ref}} \equiv 12\mu L \dot{m}_{\text{ref}}/(W_i H_o^3 \rho_{\text{ref}})$, and $\dot{m} = \dot{m}_{\text{ref}}$, i.e., $\dot{m}^* = 1$, the mass flow rate correlation with pressure difference across the channel can be elicited as

$$\dot{m} = \left(\frac{\tau_0 - 1}{2\kappa_T \ln \tau_0}\right)\left(\frac{W_i H_0^3 \rho_{\text{ref}}}{12\mu L}\right)\left((1 + \kappa_T \Delta p)^2 - 1\right). \tag{d}$$

As a reminder, ρ_{ref} is the fluid density at standard pressure and temperature. Using Eq. S(d), we can calculate a rectification ratio η as follows:

$$\eta \equiv \frac{\dot{m}_{\text{Nozzle}}}{\dot{m}_{\text{Diffuser}}} = \frac{\dfrac{\tau_{0,N} - 1}{\ln \tau_{0,N}} \times W_{i,N}}{\dfrac{\tau_{0,D} - 1}{\ln \tau_{0,D}} \times W_{i,D}}\ , \tag{e}$$

where $W_{i,N}$ and $W_{i,D}$ refer to widths at the inlet of a nozzle and a diffuser, respectively. Similarly, $W_{o,N}$ and $W_{o,D}$ denote the width at the outlet section for the fluid flow in nozzle and diffuser directions, respectively. The width profile ratios for the nozzle and the diffuser are $\tau_{0,N} = W_{o,N}/W_{i,N}$ and $\tau_{0,D} = W_{o,D}/W_{i,D}$, respectively. Note that the nozzle inlet and the diffuser outlet are the same (the nozzle outlet and the diffuser inlet are the same as well), i.e., $W_{i,N} = W_{o,D}$, $W_{i,D} = W_{o,N}$, and $\tau_{0,N} = 1/\tau_{0,D}$. Eq. S(e) becomes

$$\eta = \frac{\tau_{0,N} - 1}{1 - \tau_{0,D}} \frac{W_{i,N}}{W_{i,D}} = \frac{\tau_{0,N} - 1}{1 - 1/\tau_{0,N}} \frac{1}{\tau_{0,N}} = 1, \tag{f}$$

which states there is no flow rectification for Newtonian fluids.

The relation for a straight channel, i.e., $\tau_0 = 1$, can be obtained through $\lim_{\tau_0 \to 1} \dot{m}$, resulting in

$$\dot{m} = \left(\frac{W_i H_0^3 \rho_{\text{ref}} \Delta p}{12\mu L}\right)\left(1 + \frac{\kappa_T}{2}\Delta p\right), \tag{g}$$

which is in an agreement with our earlier work investigating the effects of channel deformability and fluid compressibility simultaneously in a rigid straight channel [23].

C.2 Extremely large flexibility parameter ($\chi \gg 1$)

For deformable channels under sufficiently large pressure differences, the flexibility parameter can be extremely large, so that the term with the third order of flexibility parameter, i.e., χ^3, dominates the left side of Eq. 4 simplifying the ODE into

$$\alpha_3 \tau^{13}(\xi)(1 + p^*(\xi)\kappa_T^*) p^{*3}(\xi) \frac{dp^*(\xi)}{d\xi} = 1, \tag{h}$$

$$\text{Initial value:} \qquad \text{at } \ \xi = 0, \quad p^* = 0.$$

For a nozzle/diffuser, where $\tau(\xi) = \tau_0 + (1 - \tau_0)\xi$, one can rearrange Eq. S(h) as

$$\begin{gathered} \alpha_3 \tau^{13} (1 - \tau_0)(1 + p^* \kappa_T^*) p^{*3} \frac{dp^*}{d\tau} = 1, \\ \text{Initial value:} \qquad \text{at } \ \tau = \tau_0, \quad p^* = 0. \end{gathered} \tag{i}$$

The analytical solution can be then written as

$$\left(\frac{\kappa_T^*}{5} p^{*5}(\xi) + \frac{1}{4} p^{*4}(\xi) \right) = \frac{\tau^{12}(\xi) - \tau_0^{12}}{12 \tau_0^{12} \tau^{12}(\xi) \alpha_3 (1 - \tau_0)}. \tag{j}$$

Because of the difficulties associated with finding the roots of the fifth-polynomial above, we consider the special case of incompressible flows ($\kappa_T \to 0$) to obtain the following analytical expression for the pressure distribution within the channel:

$$p^*(\xi) = \left(\frac{\tau^{12}(\xi) - \tau_0^{12}}{3 \tau_0^{12} \tau^{12}(\xi) \alpha_3 (1 - \tau_0)} \right)^{\frac{1}{4}}. \tag{k}$$

The dimensionless pressure at inlet, where $\tau(\xi = 1) = 1$, can then be obtained as

$$p_i^* = \left(\frac{1 - \tau_0^{12}}{3 \ \tau_0^{12} \ \alpha_3 (1 - \tau_0)} \right)^{\frac{1}{4}}. \tag{l}$$

Since $\alpha_3 = \frac{1024}{3003} (\Delta p_{\text{ref}} W_i^4 / 384 D H_0)^3$, $\Delta p = p_i^* \Delta p_{\text{ref}}$, and $\Delta p_{\text{ref}} = 12 \mu L Q_{\text{ref}} / (W_i H_0^3)$, the volumetric flow rate correlation with pressure difference across the channel, in the case of incompressible flows, can be elicited as

$$Q = \left(\frac{1}{664{,}215{,}552} \frac{(1 - \tau_0)\tau_0^{12}}{1 - \tau_0^{12}} \frac{W_i^{13}}{\mu L D^3} \right) \times \Delta p^4, \tag{m}$$

which shows a height-independent characteristic behavior, because the membrane deformation is significantly larger than the original height of microchannel under this regime. The relation for a straight channel, i.e., $\tau_0 = 1$, can be obtained through $\lim_{\tau_0 \to 1} Q$, resulting in

$$Q = \frac{1}{664{,}215{,}552} \times \frac{W^{13}}{12\mu L D^3} \times \Delta p^4, \tag{n}$$

which agrees with our other work investigating the channel deformability effects on characteristics of incompressible flow through straight microchannels [ref]. Using Eq. S(e), the rectification ratio of incompressible flows through a nozzle/diffuser channel under this regime is calculated as follows:

$$\begin{aligned} \eta = \frac{Q_{\text{Nozzle}}}{Q_{\text{Diffuser}}} &= \frac{\dfrac{(1-\tau_{0,N})\tau_{0,N}^{12}}{1-\tau_{0,N}^{12}} \times W_{i,N}^{13}}{\dfrac{(1-\tau_{0,D})\tau_{0,D}^{12}}{1-\tau_{0,D}^{12}} \times W_{i,D}^{13}} \\ &= \frac{\dfrac{\left(1-\tau_{0,N}\right)\tau_{0,N}^{12}}{1-\tau_{0,N}^{12}}}{\dfrac{\left(1-\tau_{0,D}\right)\tau_{0,N}^{-12}}{1-\tau_{0,N}^{-12}}} \times \frac{1}{\tau_{0,N}^{13}} \\ &= 1. \end{aligned} \tag{o}$$

D. Dependence on the original channel height, $\boldsymbol{H_0}$

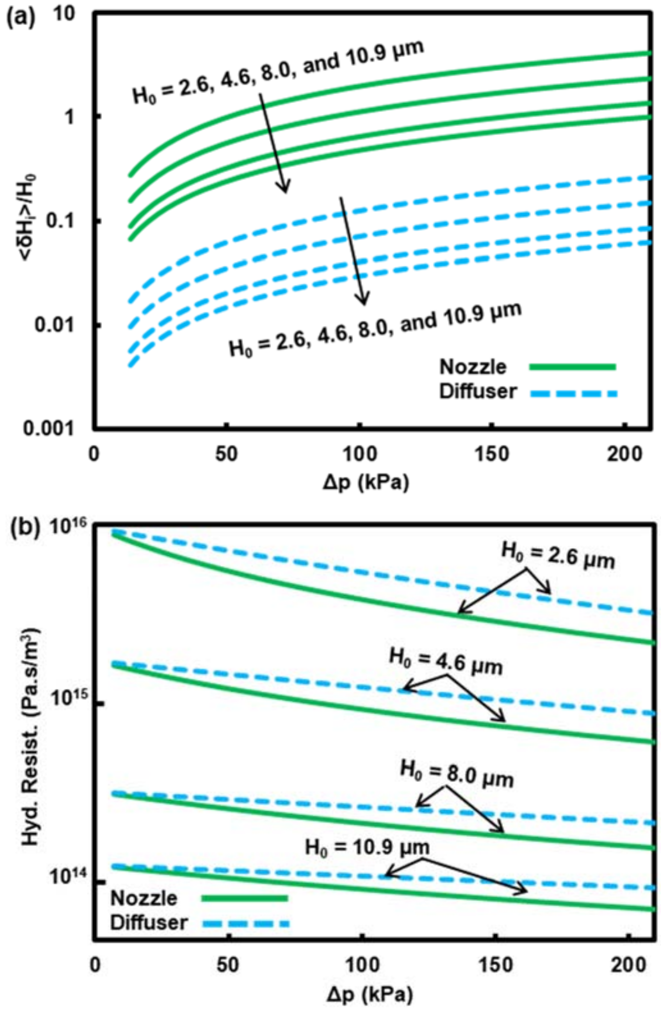

Figure S4. (a) Average of the normalized membrane displacement ($\delta H/H_0$) at inlet and (b) the hydrodynamic resistance, *i.e.*, $R_{\text{hyd}} = \Delta p/Q$, as a function of applied pressure difference (Δp) for various original channel heights (H_0). The calculation used here is based on the coupled fluid-solid mechanics model across the deformable nozzle/diffuser microchannels with the half-angle of $\theta = 1.25°$ and small and large widths of 1 and 2 mm.

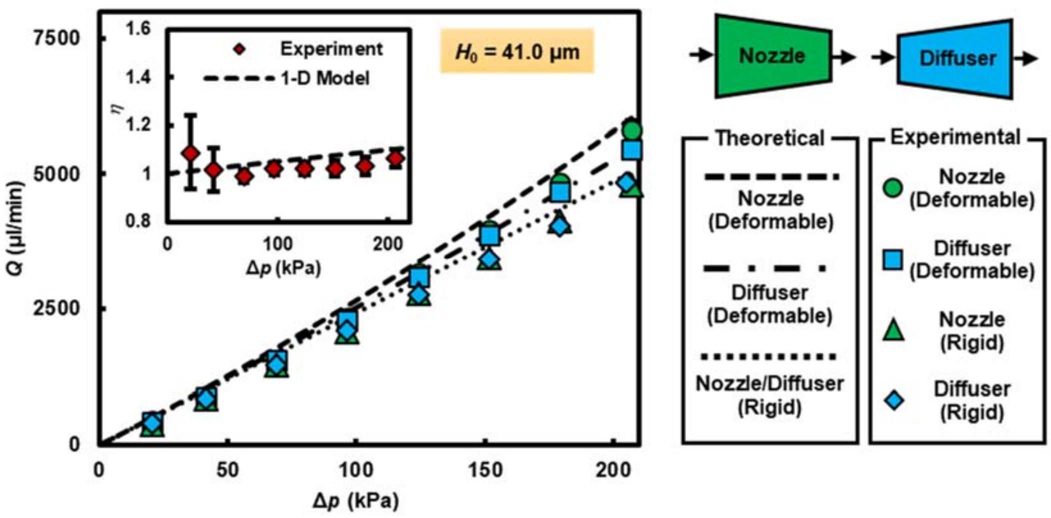

Figure S5. A volumetric flow rate (Q) of DI water as a function of pressure difference (Δp) across the deformable nozzle/diffuser microchannel of ~ 41 μm in height, small and large widths of 1 and 2 mm, and half-angle of $\theta = 1.25°$. The dashed, dash-dotted, and dotted lines show the results obtained from the fluid-solid coupled model for the deformable nozzle, deformable diffuser, and rigid nozzle/diffuser, respectively. The inset graph shows a rectification ratio, η, as a function of Δp.

E. Compressible flows in deformable shallow channels

The first-order nonlinear ordinary differential equation derived in Eq. 4 is applicable for both compressible and incompressible flows. As a special case of compressible fluid flows, an ideal gas flow is of our interest because air can be treated as an ideal gas and used in our experiments for comparison. The isothermal compressibility for the ideal gas can be written as $\kappa_T|_{p_{\mathrm{atm}},T_{\mathrm{atm}}} = 1/p_{\mathrm{atm}}$ or $\kappa_T^*|_{p_{\mathrm{atm}},T_{\mathrm{atm}}} = 1/p_{\mathrm{atm}}^*$ in a dimensionless form. Then Eq. 4 becomes

$$\left(1+\alpha_1\tau^4(\xi)p^*(\xi)+\alpha_2\tau^8(\xi)p^{*2}(\xi)+\alpha_3\tau^{12}(\xi)p^{*3}(\xi)\right)\left(1+\frac{p^*(\xi)}{p_{\mathrm{atm}}^*}\right)\tau(\xi)\frac{dp^*(\xi)}{d\xi}=1 \tag{p}$$

$$\text{Initial value:} \qquad \text{at } \ \xi=0, \quad p^*=0,$$

At the ambient temperature (295 K) and pressure (101 kPa), the dynamic viscosity and specific gas constant for air are $\mu = 1.8\times10^{-5}$ Pa $\cdot$ s and $R = 287$ J/(kg $\cdot$ K), respectively. Figure S6 shows the mass flow rates ($\dot{m}$) of the air flow through *rigid* nozzle/diffuser microchannels with various original H_0. Unlike the incompressible flow that exhibits the linear relationship between $\dot{m}$ and Δp, $\dot{m}$ of the compressible flow increases nonlinearly with Δp through the rigid microchannels. Yet the rigid nozzle and diffuser exhibit the same characteristic behavior resulting in no flow rectification, i.e., the hydrodynamic resistance independent of the flow direction.

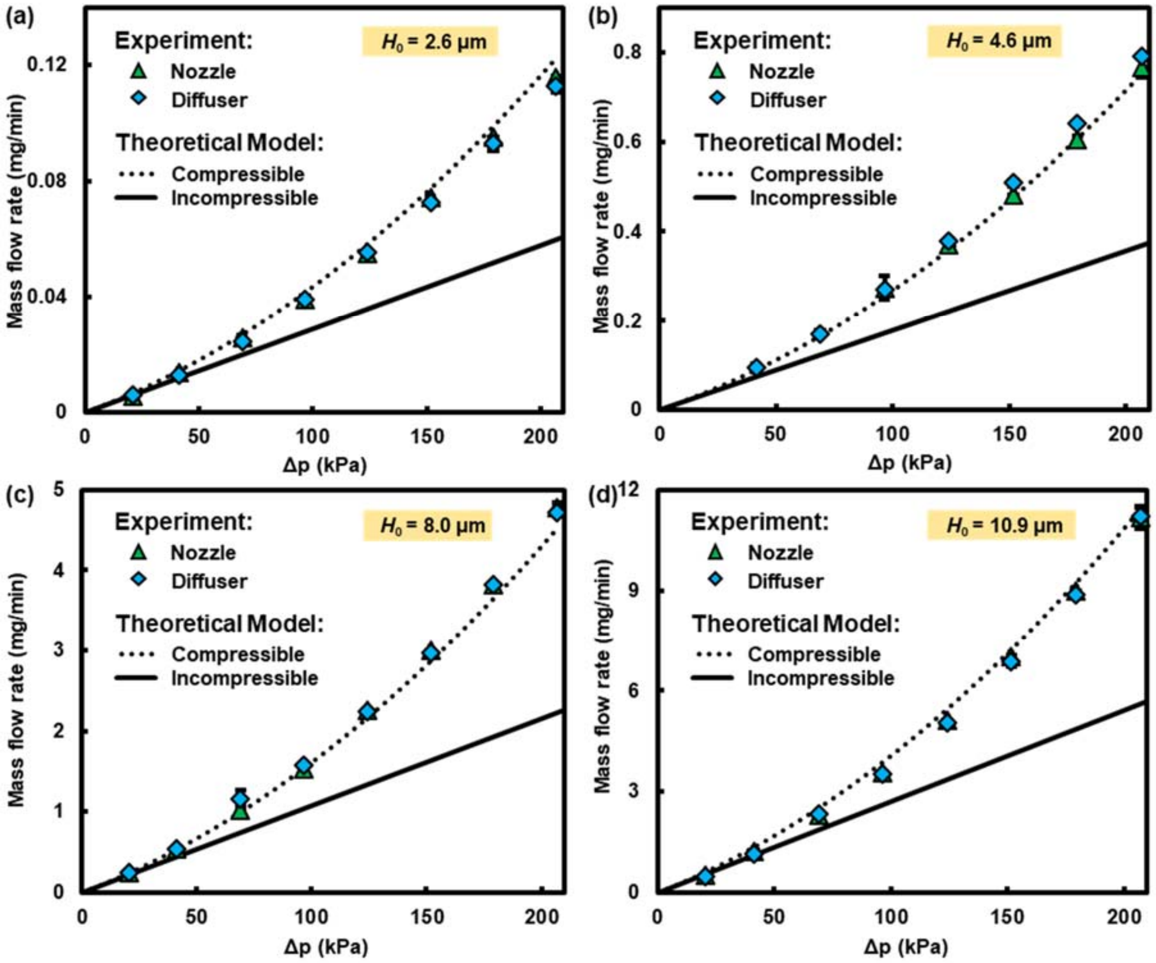

Figure S6. Mass flow rates ($\dot{m}$) of air as a function of pressure difference (Δp) across the **rigid** nozzle/diffuser microchannels with small and large widths of 1 mm and 2 mm and a half-angle of 1.25° for various original heights of H_0 = (a) 2.6 μm, (b) 4.6 μm, (c) 8.0 μm, and (d) 10.9 μm. The modeling results with and without compressibility effects taken into account are shown by dotted and solid lines, respectively.

For the case of deformable microchannels, however, a nozzle delivers a larger $\dot{m}$ than a diffuser under the same Δp, resulting in flow rectification for all four microchannels with different H_0 (see Figure S7). Just as the rigid microchannels of Fig. S6, the theoretical model that neglects the fluid's compressibility underpredicts $\dot{m}$.

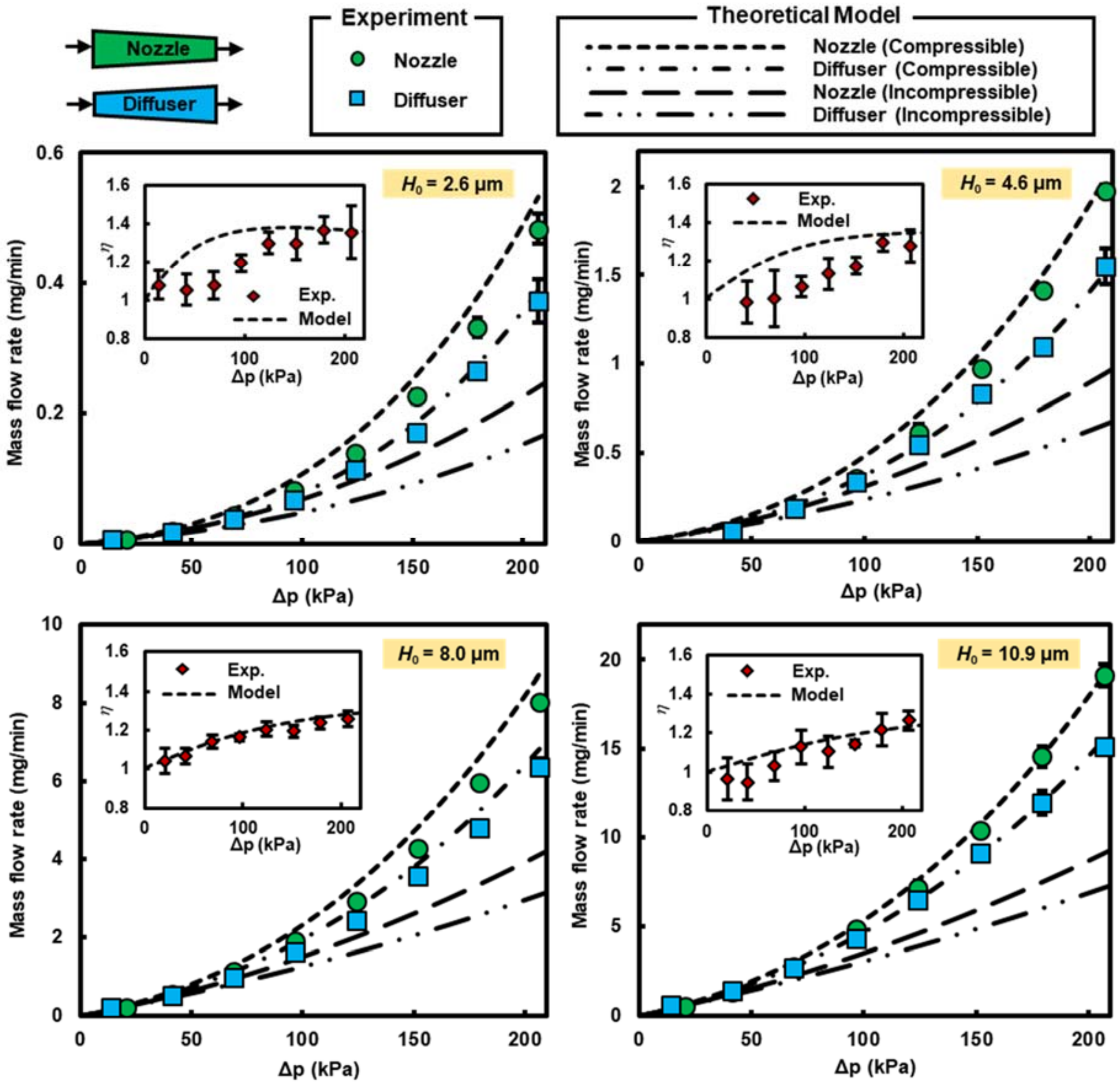

Figure S7. Mass flow rates ($\dot{m}$) of air as a function of pressure difference (Δp) across the **deformable** nozzle/diffuser microchannels with small and large widths of 1 mm and 2 mm and a half-angle of 1.25° for various original heights of H_0 = (a) 2.6 μm, (b) 4.6 μm, (c) 8.0 μm, and (d) 10.9 μm.
